# Chronic Social Stress and Susceptibility to Concentrated Ambient Fine Particles in Rats

**DOI:** 10.1289/ehp.0901631

**Published:** 2010-03-01

**Authors:** Jane E. Clougherty, Christina A. Rossi, Joy Lawrence, Mark S. Long, Edgar A. Diaz, Robert H. Lim, Bruce McEwen, Petros Koutrakis, John J. Godleski

**Affiliations:** 1 Department of Environmental Health, Harvard School of Public Health, Boston Massachusetts, USA; 2 Rockefeller University, New York, New York, USA

**Keywords:** chronic stress, concentrated ambient particles (CAPs), differential susceptibility, effect modification, respiratory function

## Abstract

**Background:**

Epidemiologic evidence suggests that chronic stress may alter susceptibility to air pollution. However, persistent spatial confounding between these exposures may limit the utility of epidemiologic methods to disentangle these effects and cannot identify physiologic mechanisms for potential differential susceptibilities.

**Objectives:**

Using a rat model of social stress, we compared respiratory responses to fine concentrated ambient particles (CAPs) and examined biological markers of inflammation.

**Methods:**

Twenty-four 12-week-old male Sprague-Dawley rats were randomly assigned to four groups [stress/CAPs, stress/filtered air (FA), nonstress/CAPs, nonstress/FA]. Stress-group animals were individually introduced into the home cage of a dominant male twice weekly. Blood drawn at sacrifice was analyzed for immune and inflammatory markers. CAPs were generated using the Harvard ambient particle concentrator, which draws real-time urban ambient fine particles, enriching concentrations approximately 30 times. CAPs/FA exposures were delivered in single-animal plethysmographs, 5 hr/day for 10 days, and respiratory function was continuously monitored using a Buxco system.

**Results:**

Stressed animals displayed higher average C-reactive protein, tumor necrosis factor-α, and white blood cell counts than did nonstressed animals. Only among stressed animals were CAPs exposures associated with increased respiratory frequency, lower flows, and lower volumes, suggesting a rapid, shallow breathing pattern. Conversely, in animals with elevated CAPs exposures alone, we observed increased inspiratory flows and greater minute volumes (volume of air inhaled or exhaled per minute).

**Conclusions:**

CAPs effects on respiratory measures differed significantly, and substantively, by stress group. Higher CAPs exposures were associated with a rapid, shallow breathing pattern only under chronic stress. Blood measures provided evidence of inflammatory responses. Results support epidemiologic findings that chronic stress may alter respiratory response to air pollution and may help elucidate pathways for differential susceptibility.

Epidemiologic evidence suggests that chronic exposures to social stressors may influence individual susceptibility to air pollution exposures. Several large cohort studies have reported stronger effects of air pollution among persons with lower socioeconomic status (SES) (Jerrett et al. 2004; Krewski et al. 2000). Smaller cohort studies have used questionnaire instruments to examine susceptibility to air pollution by specific social stressors, such as exposure to violence (Clougherty et al. 2007), fear of violence (Clougherty et al. 2006), or perceived stress (Chen et al. 2008), which may be causal components of SES-related susceptibility (Kubzansky et al. 2000).

There is growing interest in disentangling the effects of social and physical exposures, and in exploring potential synergies among these (Clougherty and Kubzansky 2009; Gee and Payne-Sturges 2004; Morello-Frosch and Shenassa 2006; O’Neill et al. 2003; Weiss and Bellinger 2006), because social stressors (i.e., poverty, violence) and environmental exposures (i.e., traffic-related pollution) may be spatially correlated, clustered in lower-income communities, and near highways and industry (Bullard 1990; Graves 1988). Thus, the most pollution-exposed communities may also be the most susceptible.

The case of traffic-related air pollution is particularly challenging. This complex chemical mix (Schauer et al. 2006) may be spatially associated with noise (Allen et al. 2009; Davies et al. 2009) and other stressors (Wheeler and Ben-Shlomo 2005). Persistent spatial correlations among these exposures may limit the utility of epidemiologic methods alone to fully disentangle their effects, and may influence the magnitude of observed effect modification. Epidemiologic methods cannot establish directionality in interactions because pollution exposures may also influence hypothalamic–pituitary–adrenal (HPA) function and stress responsivity (Sirivelu et al. 2006). Finally, epidemiologic methods cannot identify physiologic mechanisms for differential susceptibilities (Clougherty and Kubzansky 2009). As such, toxicologic data are needed to elucidate potential interactions among stressors and pollution exposures.

Traffic-related air pollution has been linked to respiratory illness and premature mortality in adults (Dockery 1993; Pope et al. 1995) and to asthma (Moore et al. 2008) and reduced lung function (Pekkanen et al. 1997) in children. There is growing interest in understanding cardiovascular effects of traffic-related pollution (Peters 2005; Rückerl et al. 2006), identifying causal constituents (Lanki et al. 2006), and characterizing susceptible subpopulations (O’Neill et al. 2007).

Chronic psychological stress is linked with alterations in immune, inflammatory (Bierhaus et al. 2003; Steptoe et al. 2007), and endocrine function (Kiecolt-Glaser et al. 2002; Miller et al. 2002; Segerstrom and Miller 2004), including dysregulation of catecholamine production (Glaser and Kiecolt-Glaser 2005). Cytokines, particularly interleukin-6, stimulate corticotrophin-releasing hormone and HPA axis activity, increasing plasma adrenocorticotropin hormone and cortisol (Hellhammer et al. 1997; Seeman et al. 1997). Frequent glucocorticoid receptor activation may produce blunted glucocorticoid response (Miller et al. 2002) and altered nuclear factor κB signaling (Miller et al. 2008). Immune-linked inflammatory responses influence airway disease through IgE, cytokine production (Chen et al. 2003), and respiratory inflammation (Umetsu et al. 2002). Early-life stress may alter T-helper 1 (T_H_1) and T_H_2 immune cell balance (Umetsu et al. 2002) linked with childhood asthma (Miller and Chen 2007). Accumulated wear and tear, compromised immune function, and enhanced general susceptibility under chronic stress are collectively referred to as “allostatic load” (McEwen 2006; McEwen and Seeman 1999). Chronic stress and pollution may also influence common physiologic systems [e.g., oxidative stress (Fujisawa 2005), IgE production (Nel 2001)], complicating directionality in interactions. Toxicologic evidence suggests permanent alterations in HPA function with chronic stress and lead exposures (Virgolini et al. 2005, 2006), and some toxicant exposures may alter stress-related pathways (Sirivelu et al. 2006).

Rat models have proven effective in studies of social stress (Grissom 2007; Sirivelu et al. 2006), air pollution (Batalha 2002), and asthma (Kodavanti 1997) and as a standard for inhalation bioassays and fuel emissions testing (Hesterberg et al. 2005). In adult rats, there is low likelihood of pulmonary overload at these concentrations of concentrated fine particulate air pollution [concentrated ambient particles (CAPs)]. In prior studies, chronic stress has induced physiologic changes in rats (Luciano and Lore 1975). As highly social animals with known behaviors for establishing and maintaining hierarchy, rats provide an appropriate model for investigating social stress effects on health. Sprague-Dawley rats have shown moderate responses to CAPs in previous studies (Clarke et al. 1999), without exaggerated responses or need for control animals as for spontaneously hypertensive rat models.

In the present study, we developed a method for examining the effects of chronic social stress in modifying respiratory response to particulate air pollution exposures in rats. We used a rat model of social stress, and the Harvard School of Public Health (HSPH) ambient particle concentrator (HAPC) system (Sioutas et al. 1995a, 1995b) was used to deliver controlled exposures. We randomly assigned rats to four exposure groups, controlling stress and pollution exposures to better disentangle their effects on respiratory function. Finally, we examined a suite of blood-borne physiologic markers of inflammation to begin to elucidate mechanisms through which chronic stress and its physiologic sequelae may influence pollution susceptibility.

## Materials and Methods

We used a rat model of social stress to compare respiratory response to inhaled CAPs exposures between chronically stressed and nonstressed animals. Twenty-four 12-week-old male Sprague-Dawley test rats and three older male Sprague-Dawley stressor rats (retired breeders, 6 months of age), obtained from Charles River Laboratories (Wilmington, MA), were individually housed at HSPH and treated humanely and with regard to minimizing and alleviating suffering, in accordance with Institutional Animal Care and Use Committee guidelines.

### Experimental protocols

Before exposure initiation, the 24 test rats were randomly assigned to four exposure groups: stress/CAPs, stress/filtered air (FA), nonstress/CAPs, and nonstress/FA.

#### Stress protocol

Stress group animals were individually introduced into the home cage of an older male (retired breeder) for 20 min on stress exposure days, following a protocol adapted from Hotsenpiller and Williams (1996). Prior research indicates that, after breeding, males display more territorial behavior, including aggression and posturing toward intruder animals (Hotsenpiller and Williams 1996; Thomas 2007), and studies using the social dominance paradigm document physiologic stress responses in intruder rats (Luciano and Lore 1975). Stress exposures were performed at the same time each day (between 1100 hours and 1300 hours), to account for diurnal variation in stress hormones and activity levels.

Prior evidence indicated that > 80% of bites received by an intruder during stressor interactions occur to the back or hindquarters, causing little to no damage to the face or respiratory organs (Takahashi and Blanchard 1982), which could otherwise interfere with respiratory function. To ensure the physical safety of test rats, without complete removal from stressful interaction, we closely monitored every stress exposure, and we placed an escape chamber from the test animal’s home cage into the stressor animal’s cage immediately before every stress exposure. The escape chamber, a four-walled Plexiglas tube with space for only one rat, allowed the intruder to protect his body while inside the stressor cage (Takahashi 1980). Test rats were rotated among the three stressor rats to account, on average, for stressors’ varying aggressivity and to ensure that stressors did not become inured to a familiar intruder.

The stress exposure protocol was initiated 1 week before CAPs exposures to establish physiologic conditions concomitant with chronic stress. The stress exposure protocol was continued throughout the study, performed 1 day before each CAPs/FA exposure.

#### Biomarkers of stress

We explored blood-borne biomarkers to begin to identify physiologic alterations in chronically stressed animals that may account for differential susceptibilities. Blood withdrawal from live rats requires a restraint, thus causing stress (Bhattacharya and Bhattacharya 1982) and potentially obscuring between-group differences; therefore, we chose to collect blood only after sacrifice. Rats were euthanized by intraperitoneal overdose injection of sodium pentobarbital (Fatal Plus; Vortech Pharmaceuticals, Dearborn, MI) to minimize physical struggle and time to death, which could interfere with stress markers. The thorax was opened and 2–3 mL blood was obtained via cardiac puncture. Blood was collected in 2-mL Vacutainer tubes pretreated with EDTA; serum was separated by centrifuge; and all blood samples were refrigerated, safely packed, and shipped via overnight courier to IDEXX Laboratories Inc. (North Grafton, MA) for analysis of serum corticosterone and whole blood. Serum tumor necrosis factor-α (TNF-α) was determined using enzyme-linked immunosorbent assay (R&D Systems, Minneapolis, MN). High-sensitivity C-reactive protein (CRP) was determined by immunoturbidimetric assay on a Hitachi 917 analyzer (Roche Diagnostics, Indianapolis, IN) with reagents and calibrators from DiaSorin (Stillwater, MN). Organs potentially affected by chronic stress (adrenal glands, spleen, and thymus) were harvested and weighed.

#### CAPs exposure protocol

Animals were continuously exposed to CAPs or FA in a single-animal plethysmograph for 5 hr/day, at the same time on 10 exposure days per animal. Three animals from each group were exposed each day, to account on average for day-to-day variability in concentration and composition of ambient particulate matter ≤ 2.5 μm in aerodynamic diameter (PM_2.5_). Exposures were initiated at the same time each day (approximately 1000 hours) to account for diurnal variability in PM_2.5_.

Animals were exposed in individual clear polycarbonate cylinders (diameter, 10 cm; length, 18 cm), which allow monitoring during exposures. Cylinders were fitted with connectors for air delivery and flow monitoring, and connections for airflow transducers (Buxco Electronics Inc., Sharon, CT). Solid panels between chambers eliminated interaction between animals. During the first 10–20 min of each exposure, animals were active while adapting to the chambers; after acclimation, animals slept through most of each exposure period.

CAPs were generated using the HAPC system (Sioutas et al. 1995a, 1995b, 1997). The concentrator draws real-time ambient PM_2.5_ (0.1–2.5 μm) from urban air, enriching particle concentrations by approximately 30 times without substantially changing their physicochemical characteristics (Saldiva 2002; Sioutas et al. 1997). The HAPC uses virtual impaction (Loo and Cork 1988) to size-select particles using inertia; ambient air is accelerated and focused at a collection nozzle, abruptly deflecting the air flow. Only particles smaller than the impactor cut point (with lesser momentum) follow the deflected streamlines. The HAPC includes three successive impactor stages. Because the ambient mass fraction of particles < 0.15 μm in aerodynamic diameter is small (McMurry and Zhang 1990), the HAPC output contains most ambient fine particle mass. Particles with aerodynamic particle diameter > 2.5 μm are removed upstream using a modified Anderson conventional impactor as a size-selective inlet.

### Continuous measures

We measured continuous (10-min average) CAPs mass using a tapered element oscillating microbalance (TEOM; model 1400ab, ThermoFisher Scientific, Fitchburg, MA) and particle counts using a condensation particle counter (model 3022A; TSI Inc., Shoreview, MN). Black carbon (BC) was measured using a aethalometer (model AE-9; Magee Scientific Inc., Berkeley CA), which measures the optical attenuation of light on an 820-nm prefired quartz fiber filter converted using an internal conversion factor derived against elemental carbon (Allen et al. 1999; Babich 2000). Concentrations were measured at the exposure point via a dichotomous split flow into the sampling manifold or exposure chambers.

### Integrated measures

CAPs samples were collected from a sampling manifold at the exposure chamber entry at 3.0 L/min on 47-mm Teflon filters (polytetrafluoroethylene; Gelman, Ann Arbor, MI) throughout each exposure (about 5 hr); samples were analyzed gravimetrically using a Mettler MT5 precision microbalance (Mettler Toledo, Columbus, OH). BC concentrations were estimated by reflectance analysis of PM_2.5_ filters using the M43D Smokestain Reflectometer (Diffusion Systems Ltd., London, UK), and absorption coefficients were calculated using ISO (International Organization for Standarization) 9835 (Cyrys et al. 2003).

### Respiratory data collection

Continuous respiratory data were collected throughout exposure periods using a whole-body plethysmography system (Buxco Electronics Inc., Wilmington, NC). Flow through each chamber was maintained at 1.5 L/min; Buxco air flow transducers (TRD5700) were connected to the chambers and to a reference chamber to compensate for pressure changes. Each chamber was calibrated to its respective transducer using a 1.5 L/min flow. We focused on seven measures: frequency, inspiratory time (TI), expiratory time (TE), peak inspiratory flow (PIF), peak expiratory flow (PEF), tidal volume (TV), and minute volume (MV; volume of air inhaled or exhaled per minute).

### Data analysis

We used two-group *t*-tests and regression analysis using categorical group indicators to test between-group differences in end-of-study blood-borne biomarkers. Because of limited power, with only one end-of-study measurement per animal, we sought between-group differences of *p* < 0.10 and calculated the effect size stress effects on CAPs response using the following formula: (mean biomarker concentration in stress/CAPs group – mean concentration in nonstress/CAPs group) ÷ mean concentration in nonstress/CAPs group.

Respiratory response to CAPs was explored using linear models, predicting respiratory measures during 10-min exposure intervals as a function of animal age, stress exposure duration, and CAPs concentration by stress group. Continuous exposure and respiratory data were summarized into 10-min intervals. We excluded the first and last 60 min of data per day, when animal movement interfered with plethysmograph readings.

To examine the modifying effect of chronic stress on associations between CAPs and respiratory function, we constructed two models using a method similar to that of Tsaih et al. (2004), wherein the first model produces the slopes and significance of CAPs effects separately by stress group, and the second model produces the test of significance for the stress–CAPs interaction.


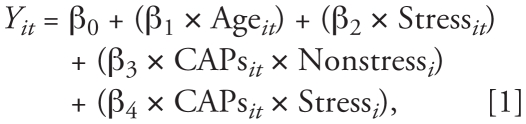


where *Y_it_* is respiratory function for rat *i* during a 10-min interval *t*; Age*_it_* is animal age in weeks at each CAPs/FA exposure; Stress*_it_* is the animal’s cumulative stress exposure in weeks, equal to zero at all times for nonstressed animals; and CAPs*_it_* is PM_2.5_ exposure during the 10-min interval *t*, equal to zero for FA animals. Stress*_i_* = 1 for stress-group animals and 0 for animals in a nonstress group. Nonstress*_i_* = 0 for animals in a stress group and 1 for those in the nonstress group. We considered effects of *p* < 0.05 significant.

A second regression model was constructed for each respiratory measure, including main effects for stress, CAPs, and their interaction:


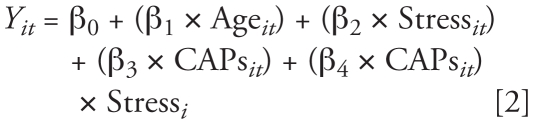


This model produces the statistical test of the interaction; if β_4_ differs significantly from zero (*p* < 0.05), then stress significantly modifies CAPs effects on respiratory function. Comparable models were constructed to examine effects of real-time BC and PM count on respiratory function.

**Figure 1:**
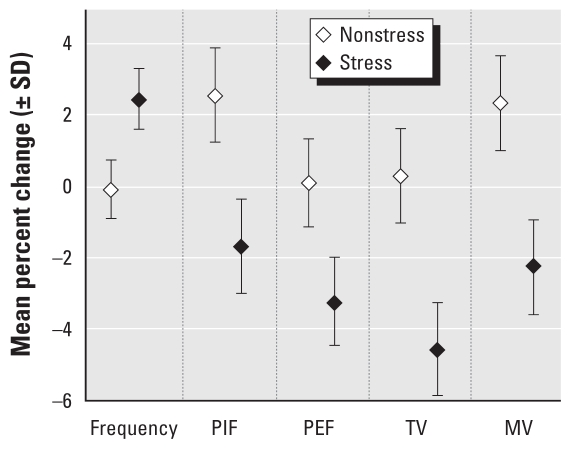
Estimated change in respiratory measures with a 1-SD (164.5 μg/m^3^) change in PM mass concentration, by stress group.

Because CAPs exposures were zero during all periods for FA animals, centering and *z*-standardization were not appropriate. Instead, we multiplied the coefficients from Equation 1 (estimated effect of 1 μg/m^3^ increase in CAPs) by observed standard deviations in CAPs concentrations (eliminating one outlier day). Figure 1 shows estimated effects for CAPs mass concentration. This method enabled comparison across CAPs measures (PM count, BC).

Before model building, we identified and removed outliers outside of mean ± 3 times the standard deviation for each respiratory and pollution measure. All analyses were performed using PROC MIXED in SAS (version 9.2; SAS Institute Inc., Cary, NC).

### Sensitivity tests

We examined sensitivity of our results to permutations in our analytic methods. First, we examined the effect of within-animal autocorrelation, owing to our repeated-measures design and small number of animals, and minor data imbalance due to missing CAPs exposure measurements. To do so, we incorporated random effects by rat (random intercepts and slopes) into linear models using PROC MIXED. Second, we repeated all analyses including only CAPs-exposed animals (*n* = 12), focusing on animals for whom CAPs exposures changed over time; this allowed us to better observe differences by stress group in response to CAPs exposure increments.

We repeated all analyses excluding one outlier high-exposure day, because FA animals may also have experienced significant gaseous exposures that day, if driven by unusual local source activity (e.g., idling diesel vehicles). Finally, we identified animals with outlier average values in any of the respiratory function measures, and we repeated the main models excluding these animals.

## Results

Sixteen successful stress exposures were performed for each animal in the two stress groups. Most stress interactions involved some physical aggression, and various defensive behaviors were observed in the test rats, including hiding, vocalizing, or adopting a submissive posture.

Twenty successful CAPs/FA exposure days were completed; each exposure day included 12 animals, 3 from each group. Thus, each animal experienced 10 exposure days, with only one exception: One rat was removed from an exposure chamber because of dehydration. Across the study period, the means ± SDs were 373.9 ± 273.8 μg/m^3^ for the 10-min CAPs mass concentrations, 1.32 × 10^4^ ± 9.93 × 10^3^ particles/cm^3^ for the CAPs count, and 10.0 ± 8.09 μg/m^3^ for BC. Continuous CAPs mass concentrations (Table 1) correlated with CAPs count (*r* = 0.77) and BC (*r* = 0.87).

Removing one outlier exposure day, continuous exposures were lower and less variable; the means ± SDs were 164.5 ± 213.2 μg/m^3^ for 10-min concentrated PM_2.5_, 5.7 × 10^3^ ± 7.2 × 10^3^ particles/cm^3^ for PM_2.5_ count, and 4.3 ± 5.6 μg/m^3^ for BC. The mean ± SD for integrated (gravimetric) ambient daily PM_2.5_ across the 20 exposure days was 9.2 ± 6.2 μg/m^3^, whereas that for integrated CAPs was 392.2 ± 249.5 μg/m^3^; ambient PM_2.5_ and CAPs were highly correlated (*r* = 0.95).

We found elevated TNF-α, white blood cell (WBC) counts, and hemoglobin in both stress groups, relative to the reference group (nonstress/FA; Table 2). CRP and absolute lymphocytes and monocytes were elevated relative to the reference group solely among stress/CAPs animals. Nonstress/CAPs exposure was associated only with elevated TNF-α. Although our small sample size may have limited power to detect significant differences, the effect sizes were notable; comparing CAPs-exposed groups, stress was associated with positive effect sizes of 0.18 for TNF-α, 0.21 for WBCs, and 0.59 for CRP.

Respiratory function measures showed substantial variability across the study and varied with animal age, CAPs exposures, and stress group. Continuous lung function measures are summarized in Table 3. All parameters vary ≥ 2-fold.

### Linear models for effects of chronic stress and CAPs on respiratory function

In linear models including main effects for stress exposures and age, with separate slopes for CAPs mass concentration exposures by stress group (Table 4), the effect of CAPs on respiratory function was qualitatively different by stress group. For stressed animals, higher CAPs exposures were associated with increased frequency, shorter TI and TE, and lower expiratory flows and TVs. For nonstressed animals, higher CAPs exposures were associated with greater inspiratory flows and MVs.

All effects of CAPs significantly differed between stressed and nonstressed animals. CAPs was associated with significantly greater increases in respiratory frequency and greater decreases in all other respiratory parameters (times, flows, and volumes) in stressed animals compared with nonstressed animals.

Figure 1 depicts estimated changes in respiratory parameters with a 1-SD increase in CAPs mass concentration (164.5 μg/m^3^), excluding the outlier day. Estimated increases in respiratory frequency are significantly greater for stressed than for nonstressed animals. For all other measures, expected decreases are significantly greater for stressed animals than for nonstressed animals.

We developed linear models to explore effects of BC exposures on respiratory response (Table 4). Among stressed animals, higher BC exposures were associated with greater frequency and lesser times, flows, and volumes. Among nonstressed animals, higher BC exposures were associated with shorter TIs but longer TEs. Estimated effects of BC on all measures except PEF differed significantly by stress group.

Comparable models were also developed to explore respiratory responses to PM count exposures (Table 4). Among animals in stress groups, higher PM counts were associated with shorter TI and TE and lower flows and volumes. Among nonstressed animals, higher PM counts were associated with greater inspiratory flows. Except for frequency and TV, all estimated effects of PM count differed significantly by stress group.

Although not a formal hypothesis, stress group differences in pollution response were generally greater with a 1-SD increase in PM count than for a comparable change in other pollution metrics. This was true for MV and inspiratory and expiratory flow. Between-group differences in frequency, TE, and TV were most evident with PM mass concentration. Differences in TI were most pronounced for BC measures.

### Sensitivity test results

We examined the sensitivity of results to permutations using analytic methods. First, we incorporated random effects into linear models to account for within-animal autocorrelation due to repeated measures. Despite limited power, results consistently supported the main results in magnitude and direction, whereas the significant effect modifications observed for frequency, TI, TE, and MV all retained significance.

Second, we repeated all analyses including only PM-exposed animals (*n* = 12), with varying CAPs exposures over time. Despite limited power, results supported the main results. Excluding the outlier exposure day did not alter observed effects of CAPs on respiratory measures. We used this more conservative range to estimate responses to a 1-SD change in concentrations (Figure 1).

Finally, we removed animals with outlier values for any respiratory measure. These were generally different animals for each measure, suggesting that no animals systematically differed. Model results generally supported the main findings, and all significant interactions remained so after excluding animals with outlier values in any respiratory parameter.

## Discussion

We found significant differences in respiratory response to CAPs and chronic stress among rats. Only under chronic stress did we observe a rapid, shallow breathing pattern; in the stress group, higher CAPs mass exposures were associated with higher frequencies and lower flows and volumes. Conversely, without stress, higher CAPs exposures were associated with greater inspiratory flows and MVs. Changes in respiratory function were comparable using CAPs count or BC exposures.

We observed changes in biomarkers indicating systemic inflammatory responses associated with airway disease among animals exposed to both stress and CAPs (Bierhaus et al. 2003; Steptoe et al. 2007). TNF-α, CRP, WBC counts, hematocrit, hemoglobin, and absolute lymphocytes and monocytes were elevated, on average, across all stressed animals relative to nonstressed animals. Compared with the reference group, CRP and absolute lymphocytes and monocytes were elevated only in animals exposed to both stress and CAPs. Stress alone was associated with elevated TNF-α, WBC and red blood cell counts, hematocrit, and hemoglobin. Nonstress/CAPs exposure was associated only with elevated TNF-α relative to the reference group.

To our knowledge, this is the first toxicologic data to examine modification by chronic stress in the effect of fine particle air pollution on respiratory function. We developed a unique experimental paradigm that provided distinction between stress and pollution exposures, often tightly confounded in real-world settings. Using this method for exploring combined effects of stress and CAPs, which can be applied to other health outcomes, we examined potential physical pathways for differential susceptibilities using blood-borne biomarkers of inflammation.

The HAPC concentrator and exposure system enabled simultaneous monitoring of real-time exposures and lung function. The system provides high exposures, well above ambient levels, over a shorter exposure time. Although the particle concentration factor decreases rapidly below 0.3 μm, ultrafine particles are concentrated. Thus, CAPs include freshly emitted traffic particles, which are mostly ultrafine but agglomerate rapidly with accumulation-mode particles. With little wood-burning in Boston, both ultrafine and accumulation BC can be a tracer of vehicle emissions. As such, although some very small traffic-related particles are not concentrated, CAPs include most traffic-generated particles.

Exposures were balanced across groups by design, because we exposed three animals per group per day, accounting on average for day-to-day variability in CAPs concentration and composition. We also initiated exposure delivery at the same time each day to account for diurnal variability in CAPs.

### Limitations

Sample size was a clear limitation; future studies should examine trends across a larger number of animals, using longitudinal models to adjust for within-animal autocorrelation in repeated measures, as applied here in sensitivity analyses. More exposure days are needed to understand effects of longer-term stress and pollution exposures on health; currently, the critical number of days required to establish “chronic” stress physiologic conditions, or to fully capture trends in respiratory response to CAPs, remains unknown.

One challenging aspect of the study of stress–pollution interactions is the distinct chronicity of stress, because acute and chronic stress produce substantively different physiologic sequelae. Acute stress effects (e.g., corticosterone production, increased ventilation and vasodilation) may, in some cases, temporarily mask effects of chronic stress (e.g., altered immune function and susceptibility) or even some immediate effects of particulate exposures. Stress is also strongly shaped by individual perception and experience; our nonstressed animals experienced their most stressful events (i.e., removal from cage) on CAPs/FA exposure days. Because lung function measures were collected concurrently with CAPs/FA exposures, these acute responses in the nonstress group may offset some observable effects of the chronic stress paradigm and may alter short-term response to CAPs in nonstressed animals. Finally, all animals experienced the acute stress of sacrifice by injection before blood draw; thus, groups did not significantly differ in corticosterone, an acute-phase stress marker.

Despite these limitations, our results suggest stronger responses to CAPs under chronic stress, including higher respiratory frequencies and lower volumes. Our model for examining effects of chronic stress and pollution may aid future studies of multiple-exposure effects on various health outcomes (e.g., cardiovascular function) and may help to elucidate pathways through which social stressors (e.g., poverty, violence) may shape population susceptibility to pollution exposures.

## Conclusions

Epidemiologic evidence suggests that chronic exposure to social stressors may affect susceptibility to air pollution in the etiology of respiratory illness. Persistent spatial correlation between stressors and pollution exposures, however, may limit the utility of epidemiologic methods alone to fully disentangle these effects or to accurately characterize interactions. In addition, epidemiologic methods cannot establish directionality in interactions or identify physiologic mechanisms that facilitate differential susceptibilities. Accordingly, we developed a model for assigning chronic social stress exposures, more clearly elucidating the role of stress in modifying respiratory response to particulate air pollution in animals.

We found that blood-borne markers of systemic inflammation were elevated under chronic stress, including CRP, TNF-α, and WBC counts. Only under chronic stress were higher CAPs exposures associated with increased respiratory frequency, reduced flows, and reduced volumes, indicating a rapid, shallow breathing pattern. Our findings support the hypothesis that chronic stress may exacerbate respiratory response to CAPs air pollution.

## Correction

In the original manuscript published online, the middle initials for two authors were incorrect; in Table 1, the animals indicated as being from the stress/FA group were actually from the nonstress/CAP group; and in Table 4, “β-value (SE)” should have been “β-value (*p*-value).” These errors have been corrected here.

## Figures and Tables

**Table 1:** CAPs mass, PM count, and BC concentration (mean ± SD) for 10-min exposures for CAPs-exposed animals, by subgroup and exposure day.

	CAPs mass (μg/m^3^)		PM count (no./m^3^)		BC (μg/m^3^)	
Exposure day	Animals A1–A3, C1–C3	Animals A4–A6, C4–C6	Animals A1–A3, C1–C3	Animals A4–A6, C4–C6	Animals A1–A3, C1–C3	Animals A4–A6, C4–C6
1	243.3 ± 311.8	NA	4040.0 ± 5139.0	NA	3.11 ± 3.16	3.18 ± 3.24
2	128.0 ± 130.71	25.7 ± 28.5	7177.1 ± 7474.4	1901.0 ± 2100.6	5.77 ± 6.28	3.51 ± 3.85
3	87.8 ± 90.0	153.6 ± 164.2	737.8 ± 768.7	3702.3 ± 3987.5	5.95 ± 6.33	4.23 ± 4.32
4	170.2 ± 173.9	182.2 ± 183.5	3264.4 ± 3424.2	2312.3 ± 2367.9	4.54 ± 4.81	5.58 ± 5.90
5	297.4 ± 306.2	204.9 ± 230.1	5896.1 ± 6093.0	4690.4 ± 5244.0	7.19 ± 7.70	9.54 ± 10.33
6	81.4 ± 82.7	219.5 ± 223.6	2373.7 ± 2470.2	8091.4 ± 8268.7	4.96 ± 5.10	8.35 ± 8.50
7	109.5 ± 114.8	185.7 ± 191.0	3450.9 ± 3527.7	5032.0 ± 5101.5	2.13 ± 2.41	2.47 ± 2.51
8	94.0 ± 95.1	140.8 ± 172.0	2774.9 ± 2842.0	4047.5 ± 4817.0	2.05 ± 2.10	10.30 ± 10.66
9	644.2 ± 650.1^a^	248.8 ± 260.6^a^	20235.3 ± 20663.5^a^	8556.0 ± 8955.8	22.99 ± 23.41	8.89 ± 9.51
10	198.1 ± 353.1	175.1 ± 180.5	5078.1 ± 7492.4	5519.5 ± 5882.1	NA	10.18 ± 10.71

Abbreviations: A, stress/CAP group; C, nonstress/CAP group; NA, because of monitoring instrument failure, continuous data were not available for some days.

a This day was eliminated as a high outlier.

**Table 2:** Biological stress measures across exposure groups (mean ± SD; 6 animals/group).

Measure	Stress/CAPs	Stress/FA	Nonstress/CAPs	Nonstress/FA^a^	*p*-Value for difference between stress and nonstress groups
Blood					
TNF-α (pg/mL)	9.6 ± 2.2*	7.3 ± 5.8*	7.9 ± 3.1*	2.0 ± 1.6	0.06
CRP (mg/L)	499.6 ± 123.8*	340.9 ± 90.8	314.2 ± 111.1	353.6 ± 52.2	0.08
Corticosterone	140.9 ± 80.5	199.2 ± 84.9	154.2 ± 93.0	210.5 ± 146.5	0.77
WBC count (10^3^ cells/μL)	5.7 ± 0.9*	5.0 ± 1.6*	4.7 ± 0.9	3.5 ± 1.2	0.03
Red blood cell count (10^3^ cells/μL)	8.4 ± 0.5	8.6 ± 0.8	8.3 ± 0.6	8.0 ± 0.3	0.10
Hemoglobin (g/dL)	15.2 ± 0.4	15.2 ± 0.9	14.9 ± 1.3	14.6 ± 0.6	0.18
Hematocrit (%)	46.2 ± 1.3	46.4± 3.3*	44.9 ± 3.9	43.5 ± 1.8	0.07
Mean corpuscular hemoglobin concentration (g/dL)	32.9 ± 0.5*	32.8 ± 0.4*	33.2 ± 0.5	33.5 ± 0.6	0.02
Lymphocyte (%)	83.7 ± 11.8	82.8 ± 4.0	80.0 ± 13.2	82.3 ± 11.7	0.63
Monocyte (%)	4.5 ± 2.7	4.3 ± 1.0	3.2 ± 0.8	3.7 ± 1.4	0.14
Eosinophil (%)	3.0 ± 4.0	1.5 ± 0.8	2.3 ± 3.8	2.0 ± 2.3	0.94
Absolute WBCs (cells/μL)					
Neutrophils	518.8 ± 389.6	548.0 ± 198.2	666.2 ± 529.5	356.2 ± 214.7	0.88
Lymphocytes	4709.0 ± 854.0*	4129.8 ± 1427.8	3727.3 ± 1049.8	2976.7 ± 1346.9	0.04
Monocytes	258.7 ± 172.8*	218.5 ± 101.6	143.7 ± 30.2	127.8 ± 60.3	0.02

Organ weight					
Adrenal (mg)	47.0 ± 3.8	50.9 ± 8.1	51.4 ± 9.2	46.1 ± 12.5	0.96
Spleen (mg)	770.8 ± 90.4	800.4 ± 94.4	805.7 ± 34.5	804.1 ± 76.6	0.29
Thymus weight/body weight (%)	1.0 ± 0.3	1.2 ± 0.2	1.2 ± 0.2	1.1 ± 0.4	0.53

a Reference group.

* *p* < 0.1 compared with reference group.

**Table 3:** Distributions of 10-min respiratory function measures (*n* = 4,532).

Measure	Mean ± SD	Median	Minimum	Maximum
Frequency (breaths/min)	122.2 ± 49.2	106.4	58.8	323.8
TI (sec)	0.22 ± 0.05	0.23	0.06	0.36
TE (sec)	0.38 ± 0.11	0.38	0.09	0.71
PIF (mL/sec)	26.0 ± 117.2	19.8	2.7	96.0
PEF (mL/sec)	25.5 ± 115.9	20.5	2.2	87.4
TV (mL)	3.4 ± 2.3	2.8	0.2	11.0
MV (mL)	389.4 ± 260.3	299.6	21.0	1435.8

**Table 4:** Linear models for effect of 10-min exposure measures on respiratory function, by stress group.^a^

Exposure, respiratory measure	β-Value (*p*-value)		*p*-Value for interaction^b^
	Stressed	Nonstressed	
PM_2.5_ mass (μg/m^3^)			
Frequency	14.0 × 10^−3^ (0.001)	−0.41 × 10^−3^ (0.92)	0.01
TI	−30.0 × 10^−6^ (< 0.0001)	−1.71 × 10^−6^ (0.69)	< 0.0001
TE	−5.0 × 10^−5^ (< 0.0001)	0.29 × 10^−5^ (0.73)	< 0.0001
PIF	−1.9 × 10^−3^ (0.17)	3.0 × 10^−3^ (0.03)	0.01
PEF	−3.9 × 10^−3^ (0.003)	0.13 × 10^−3^ (0.92)	0.03
TV	−7.3 × 10^−4^ (0.0001)	0.51 × 10^−4^ (0.79)	0.004
MV	−0.040 (0.06)	0.042 (0.05)	0.007

BC (μg/m^3^)			
Frequency	5.4 × 10^−4^ (0.0003)	0.6 × 10^−4^ (0.68)	0.02
TI	−1.0 × 10^−6^ (< 0.0001)	−0.17 × 10^−6^ (< 0.0001)	< 0.0001
TE	−0.16 × 10^−7^ (< 0.0001)	0.636 × 10^−7^ (< 0.0001)	< 0.0001
PIF	6.0 × 10^−5^ (0.22)	8.3 × 10^−5^ (0.10)	0.04
PEF	−14.0 × 10^−5^ (0.003)	4.0 × 10^−5^ (0.42)	0.12
TV	−2.0 × 10^−5^ (0.0007)	−0.33 × 10^−5^ (0.64)	0.04
MV	−13.0 × 10^−4^ (0.096)	9.26 × 10^−4^ (0.22)	0.04

PM count (no./cm^3^)			
Frequency	15.0 × 10^−2^ (0.19)	−0.97 × 10^−2^ (0.93)	0.32
TI	−7.4 × 10^−4^ (< 0.0001)	−1.6 × 10^−4^ (0.18)	0.0006
TE	−10.0 × 10^−4^ (< 0.0001)	0.4 × 10^−4^ (0.86)	0.004
PIF	−0.05 (0.18)	0.12 (0.002)	0.001
PEF	−0.115 (0.002)	0.019 (0.61)	0.009
TV	−0.014 (0.006)	−0.005 (0.38)	0.20
MV	−1.09 (0.07)	1.60 (0.07)	0.001

a Models were adjusted for continuous animal age and cumulative stress exposure in weeks, at each CAPs/FA exposure.

b The *p*-value for interaction is derived from Equation 2 and refers to the significance for the difference in CAPs response (per μg/m^3^) by stress group.
